# Spontaneous regression of locally advanced pleomorphic dermal sarcoma of the forehead: a case report

**DOI:** 10.1080/23320885.2025.2555680

**Published:** 2025-09-03

**Authors:** Silvia Rampazzo, Andrea Pasteris, Emilio Trignano, Noemi Spissu, Vincenzo Marras, Antonio Cossu, Francesco Bussu, Corrado Rubino

**Affiliations:** a Plastic, Reconstructive and Aesthetic Surgery Training Program, University of Sassari, Sassari, Italy; b Department of Medicine, Surgery and Pharmacy, University of Sassari, Sassari, Italy; c Plastic Surgery Unit, University Hospital Trust of Sassari, Sassari, Italy; d Department of Biomedical SciencesInstitute of Pathology, University of Sassari, Sassari, Italy

**Keywords:** Pleomorphic dermal sarcoma, spontaneous regression, surgical oncology, local metastasis

## Abstract

We describe a singular case in which the patient underwent wide surgical excision of the primary lesion (Pleomorphic Dermal Sarcoma) and reconstruction with a skin graft. After seventy-five days, total clinical and radiological regression of the ipsilateral parotid and neck localizations was observed without the need for adjuvant therapy.

## Introduction

Spontaneous regression of neoplastic diseases is a rare but well-documented event. Even though very little is known about the exact mechanisms behind this phenomenon, it has been shown that several circumstances such as infections, biopsy procedures, and disruptions of the tumor microenvironment, may trigger the remission of many tumors [1]. While carcinomas constitute the majority of spontaneous remission cases reported in the literature, such occurrences rarely happen in sarcomas. We here report a rare case of spontaneous regression of an advanced pleomorphic dermal sarcoma of the fronto-temporal region after the sole excision of the primary lesion.

## Case presentation

**Figure 1.**
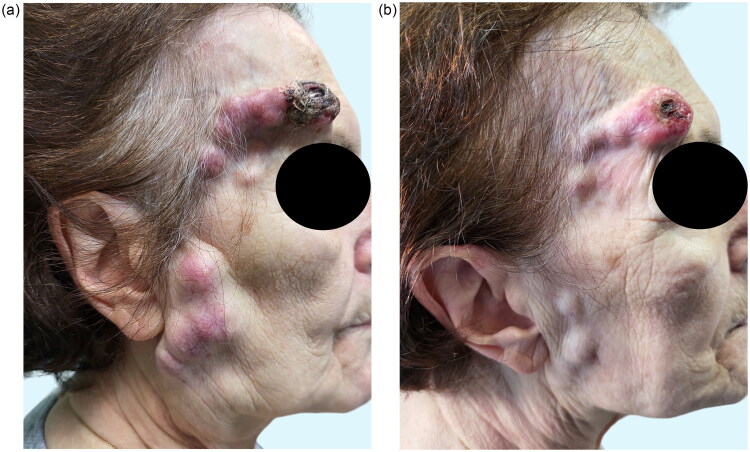
a, b) Preoperative picture of the neoplastic lesion and ipsilateral lymphadenopathy. Clinical findings at first consultation and clinical findings forty days after first consultation.

A 74-year-old woman was referred to our Unit for a rapidly growing cutaneous lesion on the right fronto-temporal region of two months duration. On physical examination, there was a multinodular, mobile, exophytic lesion of approximately 3 × 1.5 cm in dimension with overlying ulceration and hemorrhagic crust (Figure 1a). Histologic examination of the incisional biopsy, carried out during the first consultation, was suggestive for a malignant tumor of uncertain differentiation such as atypical fibroxanthoma, but histology of the whole lesion was required for a definitive diagnosis. At first consultation also multiple lymph nodes were palpable in the preauricular region, with no clinical sign of skin infiltration. Ultrasound examination, performed the day after the first consultation, revealed multiple lymph nodes suspicious for metastasis in the parotid group (dimension between 10 and 15 mm) and in level IIA (dimension 20 mm). The patient was therefore submitted to a fine-needle aspiration cytology (FNAC), at the same day, that confirmed neoplastic infiltration by highly atypical, pleomorphic, spindle and epithelioid cells with high mitotic activity. Many areas of necrosis were detectable. No immunodeficiency or hematological diseases were appreciable during preoperative blood tests. The patient reports negative past medical history, with no risk factors except for arterial hypertension under pharmacological treatment.

**Figure 2.**
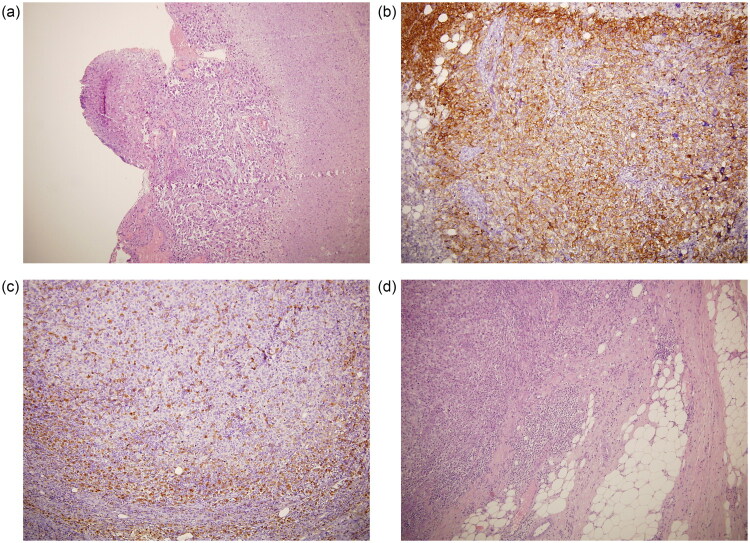
a) Histologic evaluation of the primary lesion. H&E stain showing the proliferation, at the level of the superficial and deep dermis, of atypical elements with a voluminous polymorphic and nucleolated nucleus, large eosinophilic cytoplasm with associated aspects of necrosis, ulceration of the overlying epidermis and numerous mitoses (10×). b–d: Histologic evaluation of the primary lesion. b) CD10 immunostain positivity of proliferating cells; c) CD68 immunostain positivity of proliferating cells; d) H&E stain showing poorly differentiated and solid neoplasm characterized by cells with a hyperchromic polymorphous nucleolated nucleus, intensely eosinophilic cytoplasm, often in mitosis (10×).

**Figure 3.**
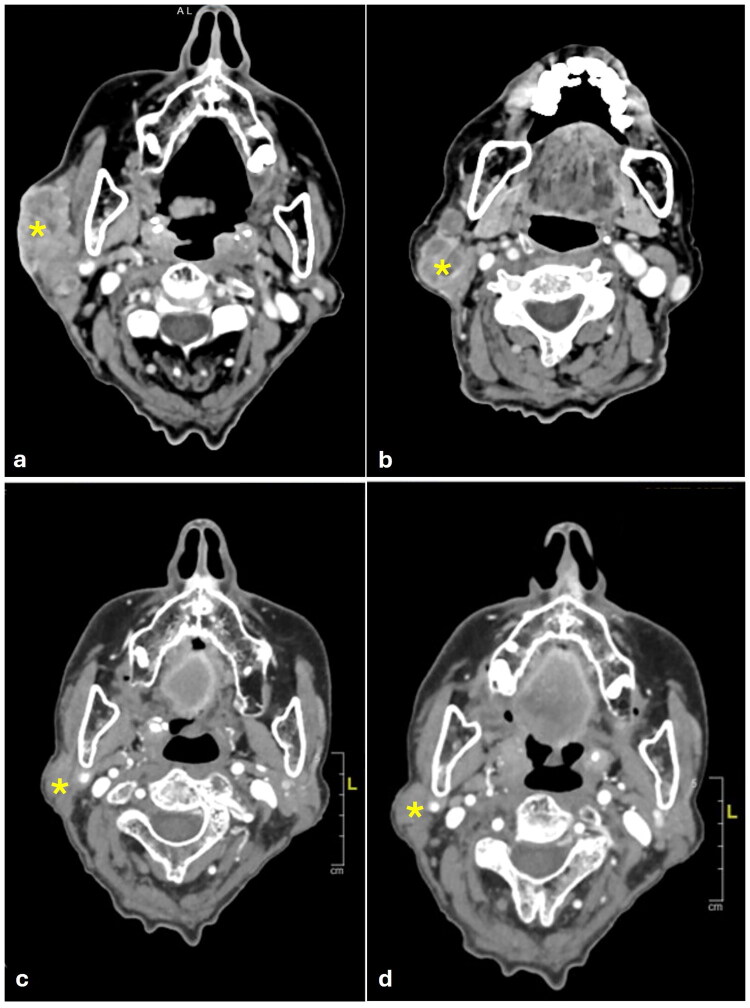
a–d: Head and neck CT scan. a) The yellow asterisk points an lesion with heterologous tissue components inseparable from a colliquative lymphadenopathy package for a maximum overall extension of 80 × 80 × 40 mm at the level of the right parotid lodge; b) The yellow asterisk points a rounded lymphadenopathy, approximately 8 mm in maximum diameter, at the right level IIB. c) The yellow asterisk points the thickening of the skin surface with modest retractive effects and obliteration of the adipose surface at the site of the lesion previously reported lesion in the right parotid lodge; d) The yellow asterisk points the thickening of the skin surface at the site of the previously reported lymphadenopathy in the IIB level.

**Figure 4.**
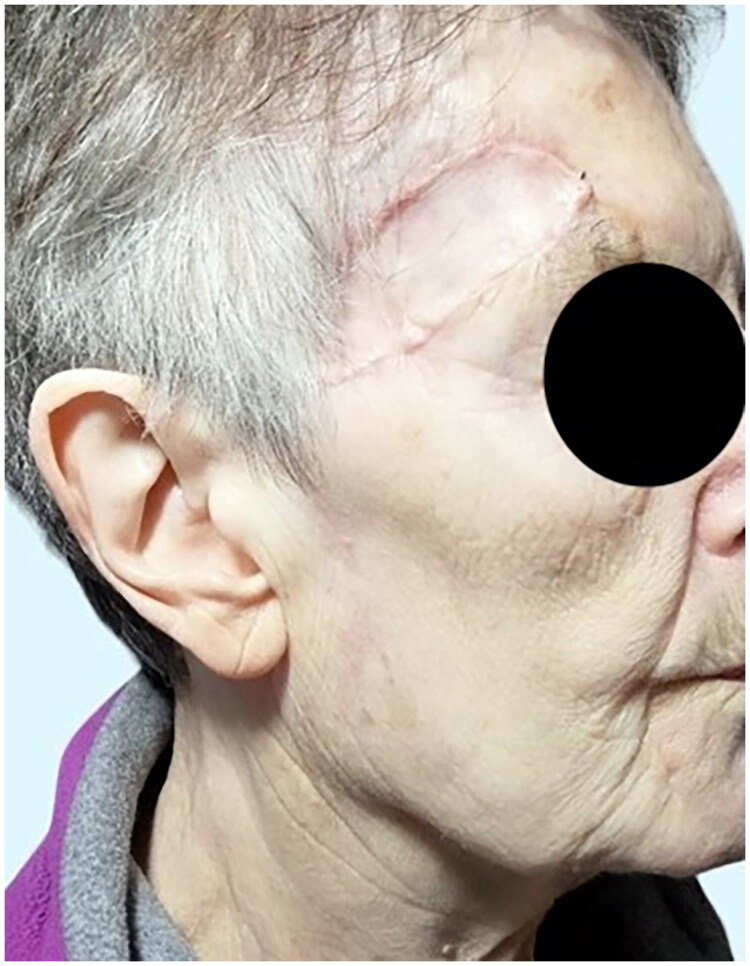
Clinical status seventy-five days after primary lesion resection. Post-operative picture that shows no sign of recurrence.

Approximately forty days after the first consultation, the patient was evaluated for surgery: the tumor had grown in dimensions and the parotid lymph nodes appeared to infiltrate the overlying skin (Figure 1b). Given the extended waiting time for a timely CT scan, the decision was made to proceed with the excision of the lesion in the interim. The patient subsequently underwent surgical removal of the primary lesion under local anesthesia, followed by reconstruction with a full-thickness skin graft harvested from the inner surface of the upper harm: macroscopically the neoplastic lesion did not infiltrate the periosteum, and a clear plane of dissection was found in the loose areolar tissue. The histologic evaluation was characterized by the presence of a poorly differentiated and diffusely ulcerated solid neoplasm, made up of cells with a hyperchromic polymorphous nucleolated nucleus, intensely eosinophilic cytoplasm, often in mitosis. Large necrotic areas were found, but no endovascular or perineural infiltration (Figure 2a). The immunohistochemical test (Figure 2b–d) showed positivity for CD10, vimentin and CD68, negativity for SOX10, ERG, AE1AE3. The findings were compatible with a pleomorphic dermal sarcoma (PDS) grade 3 according to FNCLCC. Complete resection of the neoplastic lesion was confirmed. Postoperatively, about 20 days after surgery, the skin graft was fully engrafted, with complete healing of the donor site as well and a head and neck CT scan (Figure 3a, b) was finally performed to evaluate the clinically detectable lymphadenopathy: at the level of the right parotid lodge and subauricular area a lesion with heterologous tissue components associated to polycyclic margins, adherent to the skin plane and with non-homogeneous post-contrast enhancement was found. The lesion was inseparable from a colliquative lymphadenopathy package for a maximum overall extension of 80 × 80 × 40 mm. The lesion partially incorporated the external jugular vein, while it did not have any relationship with the internal jugular vein and the carotid axis. A further solid nodulation measuring approximately 9 × 7 mm, adherent to the skin plane was seen 1 cm below the lower margin of the resection and a smaller rounded lymphadenopathy, approximately 8 mm in maximum diameter, was found at ipsilateral level IIB. No distant metastasis was found in the thorax CT scan. Multidisciplinary evaluation of the case was carried out after the patient completed all instrumental examinations: due to the involvement of multiple lymphatic levels, complete surgical resection was not guaranteed, and the patient was therefore sent for adjuvant radiotherapy. Seventy-five days after surgery, a head and neck CT scan (Figure 3c, d) was performed to define the target volume for radiotherapy: the lesion previously reported in the right parotid area was not detectable anymore, whereas a thickening of the skin surface with modest retractive effects and obliteration of the adipose surface was noticeable in the same area, as well as at the site of the ipsilateral lymph node previous location (level IIB). Regression of the lymphatic localizations was also evident clinically (Figure 4). A thorax and abdomen CT scan were also performed 90 days after surgery to complete the oncologic evaluation that showed no distant metastasis.

However, a large cystic neoformation of probable adnexal origin, measuring over 20 cm in its greatest diameter and suspicious for neoplastic nature, was incidentally discovered during follow-up in the abdomen. Adjunctive workups confirmed the diagnosis of ovarian adenocarcinoma. The patient was therefore treated with 6 cycles of neoadjuvant chemotherapy, followed by surgical resection of the ovarian tumor and adjuvant chemotherapy. No adjuvant radiotherapy was instead performed as part of the sarcoma treatment due to the radiological complete regression.

Although there are no standardized management guidelines for PDS, our Multidisciplinary Institutional Team for non-melanoma skin cancers, according to Oncology Division, decided to follow-up the patient every 6 months with ultrasound check of local lymph nodes associated to local clinical evaluation made by our Division. Total body CT scan was performed once a year with the aim of monitoring the possible development of distant metastases from the sarcoma and, in agreement with the gynecologists, also monitoring the potential occurrence of metastases from ovarian cancer.

At twenty-four months of follow-up the patient does not present any signs of clinical and radiological recurrence of the pleomorphic dermal sarcoma.

## Discussion

Atypical Fibroxanthoma (AFX) and Pleomorphic Dermal Sarcoma (PDS) tumors share many clinical, histopathologic, and oncogenic characteristics, and are likely a part of a fibrohistiocytic mesenchymal tumor spectrum [2]. For these reasons they represent an oncological and diagnostic challenge, and it’s important to differentiate them since PDS seem to be at higher risk for local recurrence and distant metastatic diseases.

The clinical presentation of PDS is usually of a rapid growing, exophytic, ulcerated and bleeding tumor. While no specific immunohistochemical (IHC) stain is indicative for PDS or AFX [3], the main defining factors of PDS include tumor necrosis, invasion beyond the superficial subcutis or vascular and/or perineural infiltration [4]. In literature it is reported that only CD10 negativity should suggest the possibility of an alternative diagnosis [5]. Treatment of such tumors still lacks evidence-based guidelines. Nevertheless, it has been demonstrated that in many cases a wide surgical excision is sufficient to reduce local recurrence, while the role of radiotherapy and chemotherapy still remain uncertain [6]. Prognosis reported in literature in patients who developed metastatic disease is poor with a median of survival between 12 and 33 months [7]. Spontaneous regression of these kinds of tumors has been reported to occur at a frequency of approximately 1 in 60,000–100,000 [8].

In our case wide surgical excision and reconstruction with an epidermal graft was performed as primary treatment, to better control local recurrence. Seventy-five days later, total clinical and radiological regression of the ipsilateral parotid and neck lymph nodes localizations was observed without the need for adjuvant therapy.

Although various mechanisms of tumor remission have been reported, this phenomenon in sarcomas is extremely rare. In Challis’s report [9], among the 504 cases with spontaneous regressions in malignant tumor, only 5 cases were sarcomas. In comparison, spontaneous regression is better documented in other skin cancers. For instance, cutaneous melanoma, particularly in its early stages, has shown relatively higher rates of regression, with histological evidence of regressed lesions seen in up to 10–35% of cases [10]. This phenomenon is often associated with immune-mediated responses, as evidenced by dense lymphocytic infiltrates and fibrosis.

Similarly, basal cell carcinoma (BCC) may undergo partial or complete regression, especially in immunocompetent patients. The mechanisms are not fully understood but may involve host immune responses or ischemic changes within the tumor [11].

Squamous cell carcinoma (SCC) also occasionally demonstrates regression, although less frequently than BCC or melanoma, and typically in association with strong local immune activity [12].

In stark contrast, spontaneous regression in PDS is virtually unreported in the literature. Only two recent cases have described regression of small, localized PDS lesions following incisional biopsy, likely triggered by immune system activation due to local trauma [13]. These cases involved minimal disease burden and did not include regional or distant spread.

Our case, however, differs substantially. The patient presented with a locally advanced PDS, involving multiple ipsilateral parotid and cervical lymph nodes, confirmed through imaging studies. Wide surgical excision of the primary cutaneous tumor was performed, followed by reconstruction with an epidermal graft, with no adjuvant therapy administered. Remarkably, 75 days after surgery, complete clinical and radiological regression of the previously involved lymph nodes was documented. This phenomenon occurred without systemic therapy, representing, to our knowledge, the first reported case in the literature of spontaneous regression of regional lymph node metastases in PDS.

The underlying mechanism of this regression remains speculative. It is possible that surgical removal of the primary tumor triggered a systemic immune response, possibly by eliminating an immunosuppressive tumor microenvironment or by releasing tumor antigens that promoted anti-tumor immunity. Alternatively, local ischemic changes or inflammation induced by surgery could have contributed to metastatic regression.

Despite the intriguing nature of this observation, a major limitation of this case is the lack of histopathological confirmation of lymph node regression, which was inferred solely based on clinical examination and imaging studies. No biopsy or surgical excision of the lymph nodes was performed post-regression to validate the resolution histologically.

Nevertheless, this case highlights a rare and unexpected phenomenon that could open new perspectives in the understanding of tumor-host interactions in pleomorphic dermal sarcoma. It underscores the potential role of immune mechanisms in tumor regression and warrants further investigation into the biological behavior of PDS and its possible responsiveness to immunomodulatory stimuli.

## Conclusion

A rare spontaneous regression of PDS was observed in a 74-years-old woman. The remarkable regression of lymph node involvement post-surgery without adjuvant therapy emphasizes the need for further research into the mechanisms of spontaneous regression and its potential therapeutic implications. This case contributes to the limited literature on spontaneous regression in sarcomas and underscores the importance of vigilant follow-up in managing such rare occurrences.
